# On the Influence of Age and Season on the Frequency and Duration of the Diseases of Adult Males

**Published:** 1842-07

**Authors:** 


### ART. IX.

*Quid faciant Ætas annique tempus ad frequentiam et diuturnitatem Morborum Hominis adulti, Inquisitio Medico-statistica.* Auctore E. FENGER, L. M. Univ. Havn.—*Havniæ*, 1840. 8vo, pp. 71.

*On the Influence of Age and Season on the frequency and duration of the Diseases of adult Males.* By E. FENGER.—*Copenhagen*, 1840.

THE importance of all topics connected with public hygiène becomes more appreciated as each succeeding year adds to our stock of information on the subject. The Registrar-general's Reports on the mortality and fatal diseases in civil life, and the Statistical Reports on the Health of the Army and Navy, have recently furnished very extensive data for the elucidation of many controverted points in medical science; but as the former of these refers generally to all classes, and the latter to persons whose occupation and habits differ materially from those of civil life, no accurate deductions can be drawn from them on a subject of vast importance in a commercial country, viz. the health of the labouring classes.

Considering the large bodies of workmen employed by government and various public companies throughout the kingdom, there should be no great difficulty in supplying this desideratum, provided the superintending medical officers of these establishments would carefully register the cases of sickness, and keep accurate returns of the number and ages of those employed. Some interesting information on this subject, obtained from the records of the East India Company's labourers in London, and the principal government dockyards, was published in the supplementary report of the factory commissioners; and we have now to notice a valuable contribution of a similar nature, on the health of the workmen in the royal naval dockyard at Copenhagen, by Dr. Fenger, the medical superintendent of that establishment, a production which does great credit to the industry and discrimination of the author. The object of the present article is to bring the facts adduced by him into comparison with such data as we possess regarding the same classes of workmen in this country; and we hope that our notice may excite the attention of those who possess similar opportunities of collecting information and induce them to make the results of their observations known to the public. Before entering on this comparison, however, it will be necessary to say a few words regarding the description of persons over whom Dr. Fenger's investigation has extended.

The workmen in the royal naval dockyard at Copenhagen are reared at the public expense. Boys are selected at the age of ten, and sent to school, where they are instructed in reading, writing, arithmetic, and the principles of religion. At fifteen they begin their apprenticeship in the dockyard, and on its completion are, at about the age of twenty, enrolled among the workmen, and thenceforth receive pay. They are principally carpenters, smiths, ropemakers, and sailmakers. After twenty years' service they are entitled to their discharge, which, however, is seldom demanded. At the end of this period, unless rendered unfit by vice or negligence, they are, if they wish it, again enrolled; and when at length, from age or disease, they have become unfit for labour, they are transferred to an invalid establishment, and employed in any light work they may be able to perform about the dockyard.

These men are under the same discipline as soldiers, and are governed by military law. They are well paid, housed, and fed. The part of the city in which they reside is appropriated exclusively to themselves; their houses are all similar, being built of stone, two stories high, and occupied by two families who have each two rooms and a kitchen; the upper story has a wooden and the lower a stone floor; to each house is attached a yard, but none have cellars underneath them, which makes the ground floor damp in some cases; the streets are wide, straight, and always kept clean. The men are visited at least once a month by their officers, who see that the houses and streets are clean and in good order. Their food is ample and of the best quality, consisting of flour, peas, meat, butter, &c. They receive also a sufficient quantity of firewood. Their working hours are from daybreak to four p.m., with an interval of half an hour; but from the 19th December to the 20th January they do not attend at the dockyard at all. The men are stated to be rather small, not very muscular, and the muscles of their lower extremities in particular badly developed; most of them marry young, and many are of very dissipated habits. Their number averages about 1240. If attacked by sickness they are sent to the hospital, where they remain till sufficiently recovered to resume their labour; but as a small sum is stopped from their pay, many are unwilling to report themselves sick, unless either unfit for work or afraid of the disease becoming serious. The name, age, trade, disease, and dates of admission and discharge or death of every patient are entered in a register; and as this has been carefully kept since 1825, it affords much valuable statistical information, which has formed the groundwork of the publication under review.

Copenhagen, to the medical statistics of which the present observations more particularly refer, is situated on the east coast of Zealand, in 55° 42′ N. lat., 12° 34′ E. long., and is divided into three parts,—the Old Town, the New Town, and the Port or Christian's Haven. The last is built on the island of Amager, between which and the rest of the town runs a narrow inlet forming the harbour. The walls of the city enclose a circuit of five miles, and the population amounts to about 116,000. The climate of Copenhagen is said to be damp and unhealthy, and the mortality to be greater than in any other town in Denmark.

The total deaths among the workmen in the royal naval dockyard during a period of eleven years (1829-39) have been as follows:

| Age. | Years of life. | Died in each period of life. | Ratio of deaths per 1000 living. | Age. | Years of life. | Died in each period of life. | Ratio of deaths per 1000 living |
|---|---|---|---|---|---|---|---|
| 15-20 | 1727 | 4 | 2·3 | 55-60 | 907 | 49 | 54· |
| 20-25 | 1738 | 7 | 4· | 60-65 | 677 | 56 | 82·7 |
| 25-30 | 1528 | 11 | 7·2 | 65-70 | 369 | 48 | 130·1 |
| 30-35 | 1509 | 15 | 9·9 | 70-75 | 296 | 47 | 158·8 |
| 35-40 | 1372 | 23 | 16·8 | 75-80 | 215 | 35 | 162·8 |
| 40-45 | 1172 | 25 | 21·3 | 80-85 | 102 | 24 | 235·3 |
| 45-50 | 1012 | 26 | 25·7 | 85-90 | 32 | 8 | 251· |
| 50-55 | 964 | 36 | 37·3 | | | | |
| | | | | Total | 13620 | 414 | 30·4 |

In the following table we shall compare this mortality with what occurs among the male population of Denmark and Sweden, and the inhabitants of Copenhagen at the same age. As these deaths, however, are stated in decennial periods, the ratio among the dockyard workmen has been recalculated, so as to include corresponding terms of life in each case.

| Age. | Ratio of deaths per 1000 living Sweden.* | Denmark.† | Copenhagen.† | Among dockyard workmen. |
|---|---|---|---|---|
| 21-30 | 9 | 9 | 11 | 6 |
| 31-40 | 13 | 12 | 20 | 14 |
| 41-50 | 19 | 20 | 35 | 24 |
| 51-60 | 30 | 31 | 53 | 50 |
| 61-70 | 58 | 55 | 85 | 100 |
| 71-80 | 117 | 107 | 148 | 173 |
| above 80 | 252 | 216 | 241 | 231 |

From this table it appears that the ratio of deaths among the dockyard labourers is lower than among the male inhabitants of Copenhagen till the age of sixty, when the reverse is observed. It is higher, however, at all periods of life than throughout Denmark or the adjacent kingdom of Sweden ; but as the population of these states is chiefly rural the excess may be accounted for by the difference which is always found to exist between the mortality of town and country districts.

As a means of comparison with a similar class of workmen in this country the following table is submitted, showing the ratio of mortality among the East India Company's labourers in London, a body of men very similar to the Copenhagen dockyard workmen, though probably neither so well fed nor housed.

| Age. | Ratio of Deaths per 1000 living among Copenhagen Dockyard Workmen. | East India Company's Labourers.‡ | Age. | Ratio of Deaths per 1000 living among Copenhagen Dockyard Workmen. | East India Company's Labourers. |
|---|---|---|---|---|---|
| 15-20 | 2·3 | not stated | 50-60 | 45·4 | 42·7 |
| 20-30 | 5·5 | 8·2 | 60-70 | 99·4 | 92·4 |
| 30-40 | 13·2 | 14·8 | 70-80 | 160·5 | 107·1 |
| 40-50 | 23·4 | 24·3 | 80-90 | 238·8 | 139· |
| | | | Total | 30·4 | 31·3 |

Thus, with the exception of the period of life from twenty to thirty, in which the Copenhagen workmen have rather the advantage, a remarkable similarity will be found to exist in the mortality up to the age of sixty, when the comparison begins to be materially in favour of the East India company's labourers. The Copenhagen workmen are described as much addicted to the use of spirituous liquors, a circumstance to which the rapid increase of mortality after the age of fifty is probably attributable, as the same feature has been observed among the older classes of soldiers in the British army at stations where intemperance greatly prevails. This point may be illustrated by the following remark in the report on the health of the troops at the Cape of Good Hope. "There seems good reason to believe that a peculiarity thus confined to the troops alone (viz. the rapid deterioration of constitution at the higher ages) may

* Mean of twenty years, 1811-30. † Mean of five years, 1832-6.
‡ M'Culloch's Statistics of Great Britain, vol. ii. Second edition. London, 1839.

in some measure be attributable to habits of intemperance, which, though they add little to the mortality of the youngest class, are likely, if persisted in, to sow the seeds of diseases which develop themselves more fully as the soldier advances in life." (p. 23.)

The extent of sickness and the diseases by which the admissions and deaths among the Copenhagen workmen have been occasioned, are shown in the following table.

| Average annual Strength, exclusive of Masters, Foremen, &c. 1100. | Annual average of diseases treated. | Annual average of deaths. | Ratio per 1000 annually. | |
|---|---|---|---|---|
| | | | Treated. | Died. |
| Fevers | 125·9 | 2·7 | 114 | 2·5 |
| Diseases of Brain and Nervous System | 17·4 | 2· | 16 | 1·8 |
| ,, Windpipe and Chest | 69·7 | 9·5 | 63 | 8·6 |
| ,, Digestive Organs | 45·7 | 4· | 42 | 3·6 |
| ,, Urinary & Genital Organs | 19· | | 17 | } |
| ,, Integumentary System | 87· | } | 79 | |
| ,, Fibrous System | 46·5 | 5·4 | 42 | 4·9 |
| ,, Eyes and Ears | 7·1 | | } 11 | |
| ,, Nose and Throat | 5· | | | |
| ,, Uncertain seat | 15· | | 14 | |
| ,, Drunkards | 8·8 | 1·3 | 8 | 1·2 |
| Wounds and Injuries | 93·6 | 1·2 | 85 | 1·1 |
| | 540·7 | 26·1 | 491 | 23·7 |

Such of the invalids as are wholly unfit for work are not sent to hospital when sick, but are attended by the medical officer in their own houses. Records of the cases among them have been kept, however, for the last four years, from which data, combined with the admissions into hospital for fifteen years, the average number of attacks occurring in one year among all the workmen has been obtained as above stated.

Before making any remarks on the relative prevalence of the different diseases among these workmen, we shall compare the extent of sickness and mortality with what occurs among similar classes in England. This comparison, however, extends only to the Copenhagen workmen under the age of fifty, as after that period of life the results are materially affected by the number of invalids. The cases of wounds and injuries have also been deducted, because they are omitted in the returns of the English dockyards.

| | Copenhagen Workmen under 50 1825-39 | Portsmouth Dockyard. 1830-32 | Plymouth Dockyard. 1829-31 | Woolwich. Dockyard. 1830-32 | Chatham Dockyard. 1830-32 | Sheerness Dockyard. 1830-32 | Pembroke. Dockyard. 1830-32 | English Dockyds. Cases of Sickness | English Dockyds. Deaths from Disease. |
|---|---|---|---|---|---|---|---|---|---|
| Strength | 12525 | 5948 | 6186 | 2233 | 3941 | 1422 | 1338 | 18835 | 15705 |
| Attacks of Sickness | 4694 | 2250 | 2145 | ...... | 1939 | 622 | 701 | 7657 | ...... |
| Deaths from Disease | 122 | 56 | 51 | 22 | ...... | ...... | 7 | ...... | 136 |
| Ratio of cases per 1000 | 375 | 378 | 347 | ...... | 492 | 437 | 523 | 406 | ...... |
| Ratio of Deaths ,, | $9\frac{7}{10}$ | $9\frac{4}{10}$ | $8\frac{2}{10}$ | $9\frac{9}{10}$ | ...... | ...... | $5\frac{2}{10}$ | ...... | $8\frac{7}{10}$ |

The similarity in the amount both of sickness and mortality in the Danish and English Dockyards is very striking, and affords an illustration of the value of such investigations. Wherever we have accurate records of the health of a body of individuals comprehending a sufficient number to obviate any casual irregularity, we may safely make deductions not only with regard to the same body of men in future, but also as to any others similarly situated. On this principle Benefit Societies have been founded, and by extending similar observations over various classes of workmen, as in the present instance, the calculations in regard to these valuable institutions may be placed on a much more accurate basis than has hitherto been found practicable.

In examining the preceding table it must be kept in view that from the 19th of December to the 20th of January the Danish workmen do not attend at the dockyard; and that many if unwell during this period remain at home, and, to avoid being placed under hospital stoppages, do not report themselves to the surgeon. Could we make the necessary allowance for that source of error, the ratio of sickness would be found almost exactly the same as the average of the English dockyards.

We shall now proceed to make a few observations on the various classes of diseases by which this mortality and sickness have been occasioned, comparing the results, where practicable, with those obtained by similar observations in this country. For this purpose we have arranged the principal diseases among the cavalry in the United Kingdom and the members of the Liverpool Friendly Society in the same form as that adopted in the work before us, in the following table.

| | Liverpool Friendly Society, 1829-30. Years of life 4615 | | Dragoon Guards and Dragoons in United Kingdom 1830-36. Aggregate strength 44611. | | | | Copenhagen Dockyard workmen. | |
|---|---|---|---|---|---|---|---|---|
| | Cases treated. | Ratio per 1000 of strength | Total cases treated. | Total deaths. | Ratio of sickness per 1000 of strength | Ratio of deaths per 1000 of strength | Ratio of sickness per 1000 living. | Ratio of deaths per 1000 living. |
| Fevers | 338 | 73 | 3444 | 66 | 77 | 1·5 | 114 | 2·5 |
| Diseases of Brain and Nervous System | 117 | 25 | 283 | 29 | 6 | ·7 | 16 | 1·8 |
| ,, Chest, &c. | 435 | 94 | 6674 | 363 | 150 | 8·1 | 63 | 8·6 |
| ,, Abdomen | 444 | 96 | 4845 | 57 | 108 | 1·2 | 42 | 3·6 |
| ,, Fibrous System | 258 | 56 | 2252 | } 112 (Fibrous System to All other diseases) | 50 | } 2·5 (Fibrous System to All other diseases) | 42 | } 7·2 (Fibrous System to All other diseases) |
| ,, Drunkards | 9 | 2 | 27 | | 6/10 | | 8 | |
| Wounds and Injuries | 526 | 114 | 5619 | | 126 | | 85 | |
| All other diseases | 392 | 85 | 18320 | | 411 | | 121 | |
| | 2519 | 545 | 41464 | 627 | 929 | 14· | 491 | 23·7 |

*Fevers.* The prevalence of fever among the Copenhagen workmen is considerably greater than among the cavalry in this kingdom or the Liverpool artisans. Of 1877 cases, 902 are stated to have been continued and 706 intermittent; the rest were most probably eruptive fevers. Intermittents are generally rare at Copenhagen, but for six years (1827-32)

that disease prevailed annually in an epidemic form, beginning in March or April and ending in July or August, for which peculiarity no cause could be assigned. Typhus has been included with common continued fever, so that there are no means of ascertaining how many cases of it occurred. The result of Dr. Fenger's observations shows that young men are most obnoxious to intermittents, and that the liability diminishes with the advance of life, while the liability to continued fever reaches its maximum between 20 and 30, and then follows the same rule. By deducting these two classes from the total nearly all the rest, supposed to be eruptive fevers, are found to occur between 15 and 30. But while the prevalence of fever diminishes with age both the mortality and intensity increase—a slight irregularity in this respect between the age of 40 and 50 arising probably from the smallness of the numbers. These results coincide with the observations made on the subject in this country by Dr. A. S. Thomson in his Statistical Inquiry on Fever. (Ed. Med. and Surg. Journal, No. 136.)

*Diseases of the brain and nervous system.* The principal diseases comprised in this class are headach, apoplexy, paralysis, convulsions, and insanity. Their prevalence among the Copenhagen workmen is greater than among the cavalry in this kingdom, but less than among the Liverpool artisans. The mortality is very high, but this is chiefly among the older men, the ratio among those under 50 corresponding very nearly with that of the cavalry who are also under that age. The proportion of cases will be found to increase considerably with age, for out of every 1000 living at the following periods of life, there were attacked annually,

| Age | 15-20 | 20-30 | 30-40 | 40-50 | 50 and upwards. |
|---|---|---|---|---|---|
| Attacked | 4 | 8½ | 18 | 15 | 29 |

The intensity of these diseases follows the same law; for while only 1 case in 24 proved fatal under 50, 1 in 5 died above that age.

*Diseases of the windpipe and chest.* Under this head we apprehend that so many other diseases are comprised with those of the lungs as materially to vitiate the accuracy of any conclusions we might draw from the preceding results; and unfortunately we possess no means of rectifying this by reference to the specific diseases whereby the admissions and deaths have taken place. Their very great prevalence and mortality among the old compared with the younger classes of workmen, in particular, induces the belief that there has been some important error in the principles of classification, as may be seen by the following results.

| | 15-20 | 20-30 | 30-40 | 40-50 | 50 & upw. |
|---|---|---|---|---|---|
| Ratio of attacks per 1000 of strength | 24 | 42 | 56 | 69 | 112 |
| Ditto of deaths per ditto | $1\frac{1}{10}$ | | $4\frac{2}{10}$ | $9\frac{3}{10}$ | $24\frac{7}{10}$ |
| Proportion of deaths to attacks | 1 in 34 | | 1 in 13 | 1 in 7½ | 1 in 5 |

This is so different from what has hitherto been observed in regard to the influence of diseases of the lungs that we fear not only have diseases of the heart been included, but also hydrothorax and other affections of a similar nature, which so often attend a breaking up of the constitution.

We are confirmed in a belief of the existence of some important error by the circumstance, that the mortality from all diseases of the chest between fifteen and thirty is lower than that arising from phthisis alone among the population at the same age of any country of which we have authentic statistical records; and the admissions into hospital are equally below the usual average. In the absence then of detailed information it were useless to speculate on the cause of this exemption, but we trust that our author will minutely investigate the subject and make known the circumstances under which it has taken place, or, if founded in error, that he will at an early period make the necessary corrections in his statement.

*Diseases of the digestive organs.* This class comprises the diseases of all the viscera contained in the abdominal cavity except the kidneys and bladder. The Danish workmen appear to enjoy very considerable exemption from diseases of this class, but which may partly arise from the slight cases not coming under medical treatment. Though they may be much less prevalent than among persons in this country, however, these diseases are of a more fatal character when they do occur, for the mortality from them among persons under fifty is almost exactly the same as among the dragoon guards and dragoons, while above that age it is much higher. The liability to them, as in the case of the diseases already investigated, increases with the advance of years, for out of every 1000 men the proportion of cases at each decennial period was

| Age | 15-20 | 20-30 | 30-40 | 40-50 | 50 and upwards. |
|---|---|---|---|---|---|
| Attacked | 18 | 31 | 38 | 43 | 68 |

The intensity of the diseases also increases with age, for the proportion of deaths to attacks under 50 is only 1 in 29, while above that period it amounts to 1 in $6\frac{3}{4}$.

*Diseases of the fibrous system.* This class corresponds very nearly with that of rheumatic affections in the military statistical reports. Under the age of 50 rheumatic affections are less prevalent among the Copenhagen workmen than either among the artisans or the cavalry, although the climate, as already stated, is very variable and damp, with frequent fogs. The influence of age on rheumatic affections is as follows:

| Ages | 15-20 | 20-30 | 30-40 | 40-50 | above 50 |
|---|---|---|---|---|---|
| Attacked per 1000 of strength at each age. | 18 | 26 | 33 | 58 | 74 |

Thus the prevalence of these affections increases regularly with the advance of age, as is the case with many of the others.

*Diseases peculiar to drunkards.* We are not aware of any disease which can be classed under this head except delirium tremens. Assuming then that nothing but this disease has been included in the Danish returns, the following comparative statement of its prevalence among the Copenhagen workmen compared with the military in the British colonies furnishes strong corroborative proof of the statement already made regarding the very intemperate habits of the former.

| | Ratio per 1000 of strength. | | Proportion of deaths to admissions. |
|---|---|---|---|
| | Admitted for delirium tremens. | Died. | |
| Dragoon Guards and Dragoons in United Kingdom | ·6 | ·09 | 1 in $6\frac{3}{4}$ |
| Gibraltar | ·7 | ·08 | 1 in $8\frac{3}{4}$ |
| Malta | ·9 | ·12 | 1 in $7\frac{3}{4}$ |
| Ionian Islands | 2·7 | ·43 | 1 in $6\frac{1}{2}$ |
| Bermuda | 8·6 | ·77 | 1 in $11\frac{1}{3}$ |
| Nova Scotia, &c. | 4·9 | ·41 | 1 in 12 |
| Canada | 4·8 | ·30 | 1 in $16\frac{1}{2}$ |
| Cape of Good Hope | ·6 | ·13 | 1 in $4\frac{1}{3}$ |
| Mauritius | 16·8 | 1·64 | 1 in $10\frac{1}{4}$ |
| Windward and Leeward command | 16·5 | 2·02 | 1 in $8\frac{1}{7}$ |
| Jamaica | 3·7 | ·81 | 1 in $4\frac{1}{2}$ |
| Ceylon | ·8 | ·16 | 1 in 5 |
| Tenasserim Provinces | 5·1 | ·73 | 1 in 7 |
| Copenhagen Workmen under 50 years | 7·3 | 1·28 | 1 in $5\frac{2}{3}$ |

From this table it appears that delirium tremens prevails to a greater extent among these workmen than among the troops in any of the British Colonies except the Windward and Leeward Command, Mauritius, and Bermuda. Nor does this excess seem to arise from the admission of many slight cases, as the intensity of the disease is greater than in any of the colonies except the West Indies, Cape of Good Hope, and Ceylon. It is also greater than in the United States, for of a series of 69 cases published by Dr. Ware of Boston, N. A., 11 died, being in the proportion of 1 in $6\frac{1}{4}$ cases.

The influence of age on this disease is very remarkable.

| Ages. | 15-20 | 20-30 | 30-40 | 40-50 | 50 and upwards. |
|---|---|---|---|---|---|
| Attacked per 1000 strength | $\frac{4}{10}$ | 3· | $7\frac{9}{10}$ | $20\frac{8}{10}$ | $10\frac{1}{10}$ |
| Died per ditto | $\frac{15}{100}$ (15-30) | | $\frac{9}{10}$ | $5\frac{1}{10}$ | 1· |

The diminution after 50 is attributed to most of the notorious drunkards having died off before that age, but it may perhaps also arise from the invalids having it more in their power to conceal their habits, and to the effects of intemperance being experienced in a less acute but no less fatal form in advanced life. This supposition is rendered probable too by the great increase in the prevalence and still more in the mortality by "diseases of uncertain seat," after 50 years of age.

*Other classes of diseases.* We have but few remarks to offer on these classes of diseases. They consist principally of syphilis and skin diseases. With regard to the first of these there is great difficulty in finding any fair standard of comparison, because the dockyard workmen rarely go into hospital for simple gonorrhœa or chancre, almost the only cases referred to under the head of "diseases of the genital organs," being such as are complicated with buboes, phymosis, or swelled testicle; whereas, among the military, even the most trifling case comes under treatment. On the other hand the members of the Liverpool Friendly Society are not entitled to medical attendance for diseases brought on by their own vices. Indeed, with regard to all the other classes of diseases also, it must be borne in mind that every case among the military appears in the returns, while many circumstances concur to prevent any but severe cases being noticed among civilians, a circumstance which must alway have a material influence on the results, and prevent their being viewed in any other light than as an approximation to the truth.

Under diseases of the integumentary system, among the Copenhagen workmen, are included 370 cases of boils and abscesses, 373 ulcers, and 309 cases of itch, leaving 210 as the amount of other diseases of the skin and glandular swellings. Almost all the "diseases of uncertain seat" occurred among the men above 50; consequently the results under that age are those which can be referred to with the greatest safety, particularly as a means of comparison with the military.

The proportion of wounds and injuries is low, forming only one fifth of the whole cases, while in the Portsmouth dockyard it amounted to one third, but at Copenhagen the workmen labour only eleven months in each year, and the amount must also be materially influenced by the relative bustle and activity in the different dockyards.

As it is of considerable importance to ascertain the duration of sickness, and the proportion constantly sick in a given number of persons, with the view of furnishing data on which the tables of benefit societies and sick clubs ought to be framed, we subjoin the following statement of the duration of each attack among the Copenhagen workmen compared with English observations on the same subject.

| Age. | Average duration of each case. | |
|---|---|---|
| | Copenhagen Labourers. | East India Company's Labourers. |
| 15-20 | 19·4 | 14· |
| 20-30 | 18·9 | 18· |
| 30-40 | 17·4 | 22·6 |
| 40-50 | 20· | 23·2 |
| 50-60 | 22·4 | 28·6 |
| 60-70 | 26·6 | 29·1 |
| 70 & upwards. | 31·4 | 31·8 |
| Age uncertain | 32·8 | |

After the age of 30 the duration of sickness is greatest among the East India company's labourers, owing probably to a smaller proportion of slight cases coming under treatment. Among the Copenhagen workmen it is materially increased between 15 and 30 by the cases of syphilis which, as already stated, seldom come into hospital till complicated with buboes, &c. thus rendering their cure very tedious; diseases of the skin also affect the results considerably at that period of life, while between 30 and 50 delirium tremens tends to diminish the general average by the rapidity with which it runs its course either to death or recovery.

The average duration of fevers of the continued type among these men has been 16 days, while that of intermittents has been $17\frac{2}{10}$. The great duration of diseases of the integumentary system arises chiefly from the cases of itch and ulcers of the legs, the former averaging $34\frac{2}{10}$ days, and the latter 31; while other ulcers are 21, and boils and abscesses $19\frac{9}{10}$ days. To those accustomed to treat itch in England this statement seems almost incredible, but it has long been known to prevail among the Danes with remarkable virulence.

The average annual "*sick time*" of each man employed in the Copenhagen dockyard was $9\frac{9}{10}$ days, but to those under 50 years of age only $8\frac{7}{10}$. Among the workmen in Woolwich dockyard it amounted to $8\frac{6}{10}$, and in Portsmouth to $7\frac{3}{10}$. Among the East India company's labourers under 50 it was $5\frac{2}{10}$, but this arose from the much smaller proportion of cases coming under treatment among them annually. We may however conclude that the sick time to each individual is rather higher among the Copenhagen workmen than among the English.

By multiplying the duration of sickness by the ratio of attacks and dividing the product by 365, we ascertain the proportion constantly sick in 1000 men to be, among the Copenhagen workmen at all ages $27\frac{1}{10}$; under 50, 24; in Portsmouth dockyard $19\frac{8}{10}$; among the East India company's labourers, at all ages $16\frac{5}{10}$, and under 50 years $14\frac{1}{10}$; while among the cavalry in the United Kingdom it amounts to about 40.

The influence of the seasons in producing disease at Copenhagen is shown in the following table.

| | Admitted into Hospital. | Treated in Quarters. | Total. | Of 1000 cases there occurred in each month Among Copenhagen Workmen.* | Of 1000 cases there occurred in each month Among the Cavalry in the United Kingdom.* |
|---|---|---|---|---|---|
| January | 483 | 21 | 504 | 63·8 | 79·1 |
| February | 669 | 16 | 685 | 95·9 | 81·8 |
| March | 710 | 11 | 721 | 91·2 | 75·2 |
| April | 767 | 7 | 774 | 101·2 | 78·7 |
| May | 720 | 10 | 730 | 92·4 | 87·9 |
| June | 720 | 11 | 731 | 95·5 | 88· |
| July | 770 | 11 | 781 | 98·8 | 89· |
| August | 776 | 10 | 786 | 99·4 | 95·5 |
| September | 587 | 5 | 592 | 77·4 | 88·6 |
| October | 560 | 11 | 571 | 72·3 | 81·7 |
| November | 558 | 12 | 570 | 74·5 | 75·2 |
| December | 288 | 9 | 297 | 37·6 | 79·3 |
| Total | 7608 | 134 | 7742 | 1000· | 1000· |

The very large proportion of admissions in April was caused by the prevalence of influenza in a severe epidemic form in 1833. If we regard this increase as accidental, June, July, and August will be found the most unhealthy both among the dockyard workmen and the troops, the latter, however, continue to suffer throughout September also. The very small number of admissions among the workmen in December and January arises of course in a great measure from the arrangement by which during one third of the former and two thirds of the latter month they do not attend the dockyards. By referring to the meteorological table given in Dr. Fenger's work, we find that the period of greatest sickness corresponds with that at which the temperature is highest, and the greatest quantity of rain falls.

The months in which the deaths took place are not stated, but of the 331 cases which ultimately proved fatal there began

| In Jan. | Feb. | March. | April. | May. | June. | July. | Aug. | Sept. | Oct. | Nov. | Dec. |
|---|---|---|---|---|---|---|---|---|---|---|---|
| 59 | 29 | 18 | 27 | 25 | 22 | 20 | 35 | 24 | 28 | 20 | 24 |

* In both calculations allowance has been made for the different length of the months.

The most striking feature in this statement is the large proportion of fatal cases which began in January, a month when the men are less under medical supervision than at any other period, and would lead to the inference of the great importance of medical treatment in the *early stages* of disease. The influence of the seasons on the intensity of disease is very different from its influence on the prevalence of disease, for there ultimately died of the cases occurring in the quarter Dec.—Feb. 7½ per cent.; March—May, 3$\frac{1}{10}$; June—August, 3$\frac{3}{10}$; and Sept.—Nov. 4$\frac{1}{10}$; consequently in the two quarters when the fewest cases occurred the largest proportion proved fatal.

It now only remains to enquire how far each class of diseases has been influenced by season. As the number of cases, however, is too small to ensure regularity by the monthly results, it becomes necessary to combine them into quarterly ones, making the requisite allowance, as formerly, for the different length of each quarter. In the following table, prepared according to this form, Dec. Jan. and Feb. have been included as winter, and June, July, August, as summer; so that the three coldest months might be brought together in the one instance, and the three warmest in the other.

| | Fevers. | Diseases of the chest. | Diseases of the abdomen. | Diseases of the genital organs. | Diseases of the Integumentary system. | Diseases of the fibrous system. | Wounds and injuries. | Other classesof diseases. | Total. |
|---|---|---|---|---|---|---|---|---|---|
| Dec. Jan. Feb. | 14·2 | 20·4 | 8·8 | 4·9 | 20·3 | 12·2 | 18·8 | 13·2 | 112·8 |
| March, April, May | 44·8 | 22·1 | 8· | 3·9 | 28·8 | 12·3 | 22·9 | 12·4 | 155·2 |
| June, July, August | 42·2 | 16· | 19·5 | 4·3 | 21·1 | 12·6 | 29·8 | 15·2 | 160·7 |
| Sept. Oct. Nov. | 27·1 | 13· | 10·3 | 7·3 | 19· | 9·6 | 24·3 | 12·8 | 123·4 |
| Mean | 32·1 | 17·9 | 11·6 | 5·1 | 22·3 | 11·7 | 23·9 | 13·4 | 138· |

It must be borne in mind that the cases recorded in the winter must, as already mentioned, have been fewer that what actually occurred, owing to a large proportion of the workmen not being under the usual strict medical superintendence. Could the necessary correction have been made for this contingency it is probable that diseases of the chest would be found most prevalent during that quarter. In the spring of 1833 influenza prevailed, which materially increased the ratio attacked, but in both the spring and winter seasons the cases are considerably above the average. The opposite holds true with respect to abdominal disease, which, as in this country, prevails to the greatest extent during the hot weather. Rheumatism has been less prevalent during autumn than at any other season, but the heat of summer does not seem more favorable to it than the changeable and damp weather of spring. The proportion of wounds and injuries is of course highest at those seasons when the working hours are longest. Fevers have prevailed most in the spring quarter, but this has depended on the epidemics of intermittent before alluded to, for the average number of cases of common continued fever has been

In winter 9·1. Spring 14·7. Summer 26·9. Autumn 14.

A result quite in accordance with observations made in this country.

The influence of the seasons upon disease, however, is materially affected by the age of the individuals, as will be seen by the following statement of the average number of cases occurring in each quarter at three different periods of life.

| | 15-30 | 30-50 | 50 and upwards. |
|---|---|---|---|
| December, January, February . | 37·7 | 32·5 | 42·3 |
| March, April, May . . | 62·7 | 50·4 | 41·9 |
| June, July, August . . . | 62·7 | 59·6 | 38·3 |
| September, October, November . | 49·6 | 41·8 | 31·7 |

Thus among the young men the greatest amount of sickness occurs in spring and summer; among the middle-aged in summer; while in the decline of life they suffer most from the severity of winter and the variable weather of spring.

In concluding our remarks on this interesting subject, we cannot but express regret that detailed abstracts of the diseases have not been published, because however valuable the conclusions arrived at by statistical investigation may be, they lose much of that value when the precise data on which they have been founded are withheld. We are moreover prevented thereby from entering upon a most interesting branch of investigation, the influence of climate upon particular diseases, phthisis for example. On the whole, however, the work before us reflects much credit on the talents and industry of the author, and sets an example worthy of imitation in this country, and which we trust will soon be followed by the medical officers in charge of the government dockyards, or of other establishments employing large bodies of workmen.

---

### Art. X.

*Guy's Hospital Reports.* No. XIII. Oct. 1841.—*London,* 8vo.

